# eMindLog: Self-Measurement of Anxiety and Depression Using Mobile Technology

**DOI:** 10.2196/resprot.7447

**Published:** 2017-05-24

**Authors:** Thomas M Penders, Karl L Wuensch, Philip T Ninan

**Affiliations:** ^1^ Brody School of Medicine Department of Psychiatry and Behavioral Medicine East Carolina University Greenville, NC United States; ^2^ East Carolina University Department of Psychology East Carolina University Greenville, NC United States; ^3^ Brody School of Medicine Department of Psychiatry and Behavioral Medicine East Carolina University Washington, NC United States

**Keywords:** mobile, anxiety, depression, internet, measurement

## Abstract

**Background:**

Quantifying anxiety and depressive experiences permits individuals to calibrate where they are and monitor intervention-associated changes. eMindLog is a novel self-report measure for anxiety and depression that is grounded in psychology with an organizing structure based on neuroscience.

**Objective:**

Our aim was to explore the psychometric properties of eMindLog in a nonclinical sample of subjects.

**Methods:**

In a cross-sectional study of eMindLog, a convenience sample of 198 adults provided informed consent and completed eMindLog and the Hospital Anxiety and Depression Scale (HADS) as a reference. Brain systems (eg, negative and positive valence systems, cognitive systems) and their functional states that drive behavior are measured daily as emotions, thoughts, and behaviors. Associated symptoms, quality of life, and functioning are assessed weekly. eMindLog offers ease of use and expediency, using mobile technology across multiple platforms, with dashboard reporting of scores. It enhances precision by providing distinct, nonoverlapping description of terms, and accuracy through guidance for scoring severity.

**Results:**

eMindLog daily total score had a Cronbach alpha of .94. Pearson correlation coefficient for eMindLog indexes for anxiety and sadness/anhedonia were r=.66 (*P*<.001) and r=.62 (*P*<.001) contrasted with the HADS anxiety and depression subscales respectively. Of 195 subjects, 23 (11.8%) had cross-sectional symptoms above the threshold for Generalized Anxiety Disorder and 29 (29/195, 14.9%) for Major Depressive Disorder. Factor analysis supported the theoretically derived index derivatives for anxiety, anger, sadness, and anhedonia.

**Conclusions:**

eMindLog is a novel self-measurement tool to measure anxiety and depression, demonstrating excellent reliability and strong validity in a nonclinical population. Further studies in clinical populations are necessary for fuller validation of its psychometric properties. Self-measurement of anxiety and depressive symptoms with precision and accuracy has several potential benefits, including case detection, tracking change over time, efficacy assessment of interventions, and exploration of potential biomarkers.

## Introduction

Quantified information is the basis of evidence in science and medicine. Objective measures are more easily validated with independent reference frames. Subjective measures, being self-referential in nature [1], have larger and variable errors in measurement. In mental health, subjective measures provide unique data but come with challenges. Measuring one’s mental experiences requires the individual to demarcate the experience to be assessed, discern through self-reflection, provide an internal context for grading, and translate the experience into a numerical quantity.

Measuring the severity of signs and symptoms of mental experience can be performed by clinician interviews or self-report by subjects, each providing unique contributions [2]. While clinician and subject ratings are generally concordant, 44% of subjects reported scores more than 1 standard deviation from the clinician-rated scores in a study of recurrent depression [3]. Outcomes differed based on the measure used, raising inferential issues. While clinicians have a reference frame from their wealth of clinical experience, self-reporting may more accurately measure subjective experiences that are difficult to articulate in words and are also less influenced by interviewer relationships.

Use of scales have traditionally been distinct from diagnostic tools, though recently, diagnostic criteria and their frequency have been combined to gauge the severity dimension [4,5]. There is value in measurements that can assess severity while also providing proxies for diagnostic thresholds. However, diagnostic boundaries in psychiatry are arbitrary and thus are a limitation as diagnostic comorbidities are the norm in psychiatric populations. An unverified assumption is that the criteria for distinguishing diagnostic differences also provide a comprehensive measure of the disorder. An additional risk is that the diagnostic criteria may change with advancing knowledge.

There are several self-report scales available for depression and anxiety. These have significant limitations [6,7], including being solely based on psychological approaches and guided by theoretical formulations (eg, cognitive theories of depression) or by treatment response characteristics (eg, tricyclic antidepressants). An alternative is a neuroscience-based approach, which broadly distinguishes affective from cognitive neurosciences [8,9], with conscious expression elementally distinguishing emotions from thoughts, with behaviors as the output. A brain circuit‒based approach, the Research Domain Criteria (RDoC) initiative, emphasizes dimensionally oriented behavioral measures, aiming to validate them for eventual clinical work [10]. RDoC distinguishes domains of positive affect from negative affect as well as cognition. The RDoC effort has so far focused on behavioral measures (tasks) and has not yet moved to the level of self-report [11]. The development of the self-report measure reported here is such an effort founded on the domain distinctions in RDoC and thus underlying neurobiological processes. The aspiration is to provide more precise measures that provide enhanced clinical value.

Depressive biotypes based on neurobiological substrates have identified neurophysiological subtypes of major depression, reflecting distinct patterns of dysfunctional connectivity [12,13]. Rating scales derived from underlying neurobiology are necessary to optimally explore such depressive biotypes at a clinical level. Dividing symptoms based on neurobiology may better explain symptom heterogeneity and divergent responses to treatment [14]. To achieve this, symptoms need to be revisited in the framework of neurobiology.

### Web-based Mobile Technology

Web-based technology has advanced to where mobile devices are used by increasingly large proportions of the population. Additionally, there is growing interest in tracking a variety of information, from measures of well-being to clinically relevant markers that can monitor variables relevant to disease management. Measurement tools that use mobile devices can provide value in behavioral health interventions [15], particularly as willingness for self-disclosure is enhanced [16]. Mobile technology also permits more frequent assessment, addressing the recency bias in less frequent assessments [17]. In the domain of anxiety and depression, there is a need for measures that capture the enormous variability of mental phenomena in multidimensional space and flowing over time. Such massive data need to be reduced to global severity scores and indexes of value for personalized medicine, case detection, tracking over time, and potentially differential responses to interventions [14].

The aim of this study was to examine the internal consistency (reliability) of a new Web-based self-report measure of anxiety and depression and its convergent validity compared to a standard reference scale. The hypothesis is that the new measure would have an acceptable Cronbach alpha (>.7) and a large Pearson correlation coefficient (>.5) with the reference scale.

## Methods

### Measure

eMindLog [18] is a self-report measure of anxiety and depression, associated symptoms, and functioning. eMindLog is administered on a mobile device or computer in a platform-independent manner. To provide clarity and enhance precision, terms used in the scale are succinctly described before the corresponding question—a unique approach in self-report measures. Accuracy of scoring by the subject is enhanced by scoring guidance. The time and burden of assessment are taken into consideration in choosing which symptoms need daily assessment (ie, they commonly fluctuate), versus those that can be reliably condensed for weekly assessment. Thus, eMindLog has two components: a daily set of items and a weekly set. A daily total score provides a global measure, useful in differentiating treatment groups and for global decisions at the individual level. Specific item scores and derivative index scores can provide granularity for various analyses, particularly for unique profiles at the individual level, necessary for personalized medicine. Algorithms can derive boundaries that reflect *Diagnostic and Statistical Manual of Mental Disorders-5* (DSM-5) diagnostic thresholds for Major Depression and Generalized Anxiety Disorder. Content validation data from a discontinued scale provided guidance for the current scale items and their descriptions.

The version of the scale used in this report had 20 questions in the daily set and 14 in the weekly set (see Multimedia Appendix 1). Based on the results reported below, 3 items in the daily set were removed. These items were subsequently restructured and added to the weekly set and the process discussed below. Thus, the final version of eMindLog has 17 items in the daily component measuring emotions, thoughts, and behaviors from which 4 indexes are derived: anxiety, sadness, anger, and lack of pleasure. The weekly component (also 17 questions) addresses associated symptoms, interpersonal relations, quality of life, and functioning.

Why are emotions, thoughts, and behaviors so critical to measure? Emotions are subjective, felt experiences that are often difficult to describe in words. Emotions are bathed in internal body states, often reflecting somatic characteristics [19]. Emotions are associative, often independent of their temporal sequence in explicit memory. Intense emotions are immediate, not deliberate or willful. Elegant experiments demonstrate that the correlates of emotions are initiated by pattern recognition before conscious awareness of their trigger [20], with a later, conscious capacity to either let them be, inhibit, or embellish them. Primary emotions such as anxiety, anger, and sadness are mediated by the threat (negative valence) system, and anhedonia by hypoactivity of the reward (positive valence) systems [20,21].

Thoughts are subjective ideas and concepts capable of being transduced into words and articulated as speech [22]. Thoughts are capable of being reasoned, logical, proportionate, and flexible to changing situations. The focus of thinking may be narrow and detailed or broad and strategic. The sequence of thoughts, when smooth, may be linked to a theme without digressions. Thinking may be active and effortful or passive and repetitive as in habitual ways of the mind or automatic thoughts [23]. Thoughts are the conscious output of the cognitive system—much of the neuroscience effort studying cognition has focused on the process of components such as selective attention, working and declarative memory, and effortful control [11]. In a self-report, the focus is on thoughts as the conscious output of cognitive systems.

The content of thoughts is tethered by emotions. Intense emotions make thoughts captive, inducing certainty or disorganization (unsettled and turbulent). When emotions control thinking, they are hard to regulate, making deliberate and considered decisions difficult. Thus, anxious emotions make thoughts worrisome, and sad feelings biases one into beliefs of failure. Thinking can flow with emotions, embellish them if they are consonant, or counter them, which requires active effort. Cognitive therapy aims to enhance the control of thoughts over emotions. Changes in beliefs and processing routines are critical for benefit with cognitive therapy.

Behaviors are observable actions. They are in response to sensory stimuli (reflexive), emotions (conditioned), and thoughts (chosen). In situations when emotions are not intense, behaviors can be autonomous, voluntary, intentional, and willfully chosen.

**Figure 1:**
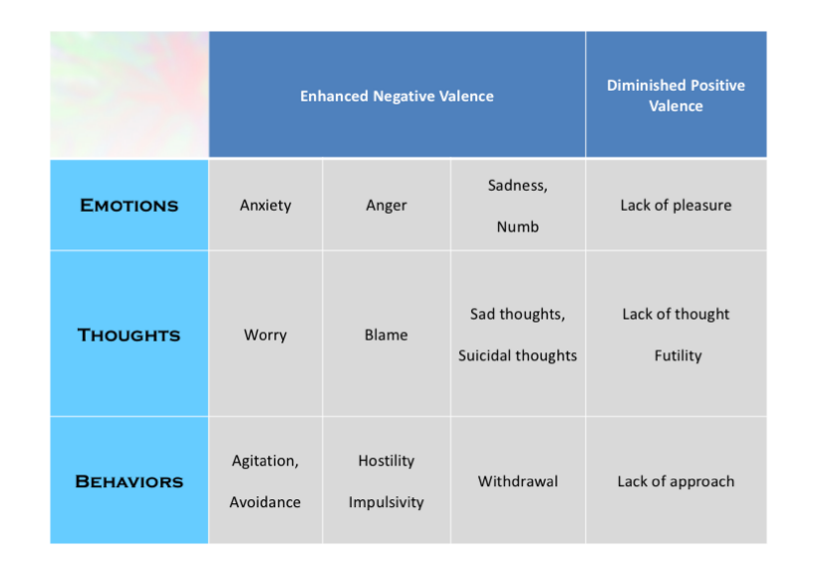
Emotions, thoughts, and behaviors - items from the threat and reward systems.

Behaviors in the repertoire of anxiety and depressive disorders are limited. To achieve higher resolution in the measurement of behaviors relevant to anxiety and depression, their motivational drivers need to be considered in parceling out the pathways to their external manifestation (see Figure 1).

eMindLog leverages mobile technology to take advantage of ease of repeated measurement and tracking ability. At a subsequent stage of development, the relationship between eMindLog scores and RDoC-based behavioral tasks can be explored.

The items in the eMindLog daily and weekly measures can be examined in an exploratory manner, to indicate potential diagnostic thresholds. For such purposes, an item is marked as present if the score is 4 or greater (moderate or above). The DSM-5 criteria for Major Depressive Disorder and Generalized Anxiety Disorder were matched with items in eMindLog in algorithms based on these criteria.

### Protocol

This validation study protocol and informed consent form were approved by the East Carolina University Institutional Review Board, and the study was compliant with the Declaration of Helsinki code of ethics. A sample of 198 English-speaking adults provided informed consent. They were a convenience sample recruited from four expedient public locations (ie, a bookstore, wellness center, psychiatry clinic staff, and college commons) on different days during September through November 2015.

The informed consent process on paper was separate from the subsequent data gathered on laptops, maintaining data anonymity. Each subject provided demographic information (ie, age, sex, race/ethnicity, socioeconomic information, and educational level) and whether they were receiving professional care (ie, psychotherapy and/or medication) for anxiety or depression. Subjects viewed a 5-minute introductory video and completed the eMindLog daily and weekly sections. Subjects also took the Hospital Anxiety and Depression Scale (HADS) [24] as a reference and answered a series of questions to provide feedback on eMindLog. The HADS was chosen as it has anxiety and depressive subscales, permitting a comparison with the eMindLog indexes. Subjects rated the eMindLog daily and weekly items, as well as the HADS on only one occasion in this study. Each received a US $25 gift card for their time and effort. An individual could participate only once.

The version of eMindLog used in the study had 20 daily questions and 14 weekly questions. The daily questions addressed anxiety (emotion: anxiety; thought: worry; behavior: physical agitation, avoidance), anger (emotion: anger; thought: blaming; behavior: hostility, impulsivity), sadness (emotion: sadness, emotionally numb; thought: sad thought, suicidal thought; behavior: withdrawal), anhedonia (emotion: lack of pleasure; thought: lack of thought, futility; behavior: lack of approach), and asocial (emotion: lack of compassion; thought: distrustful; behavior: asocial). Thus, there were 6 items related to emotions, 7 related to thoughts, and 7 related to behaviors. Each item was described (eg, “Anxiety is feeling nervous, uneasy, apprehensive or panicky”) before the question was posed (eg, “During the past 24 hours, how anxious have you felt?”). Scoring used a discretized analogue scale (0-10) enhanced with descriptive anchors and color [25]. The descriptive guidance provided was 0 labeled as none and 10 as extreme, 1-3 as mild, 4-6 as moderate, and 7-9 as severe.

The weekly questions ask the individual to rate their experiences during the past 7 days. The weekly questions were divided into 8 questions related to associated symptoms: well-being, fatigue, emotional pain, memory, concentration, appetite, sleep, and stress; a single question on quality of life; and 5 questions on functioning in the domains of social, work, school, home, and hygiene/grooming.

The scores for the daily questions were totaled for a total score and divided by the number of questions to provide a total average score. Index scores for each of the 5 indexes (ie, anxiety, anger, sadness, anhedonia, and social) were calculated as the average of 3 items including one each from emotion, thought, and behavior. When there were two items for a category (ie, physical agitation and avoidance as behaviors in the anxiety index), the higher score was used as the behavior score in calculating the index. This approach provided equivalent weighting for emotions, thoughts, and behaviors in each index. The score for functional restriction was the highest score among the 5 questions related to functioning.

### Statistical Analysis

The item scores were computed for *t* tests and analysis of variance (ANOVA). Being a study based on a general population sample, skewness was expected. One below 2 was considered acceptable by convention as too small to have a practical impact [26].

The statistical analysis assessed the internal consistency (reliability) for the eMindLog daily total score and the index scores, as well as the HADS scores. A Cronbach alpha ˃.7 is considered the threshold for acceptability.

Convergent validity of the eMindLog was assessed by examining the relationship between specific eMindLog index scores and the HADS Anxiety and Depression subscale scores. A Pearson correlation coefficient ˃.5 was considered a large strength of association.

The diagnosis of Generalized Anxiety Disorder and Major Depression are based on symptom criteria, all of which are assessed in eMindLog. The items in eMindLog daily and weekly were matched to DSM-5 diagnostic criteria for Major Depressive Disorder (MDD) and Generalized Anxiety Disorder (GAD) using an exploratory algorithm. Since this study was a one-time assessment, the time requirements for MDD (2 weeks) and GAD (6 months) could not be considered, though the requirement for impaired functioning was included. To assess whether this approach was valid, a sensitivity analysis was performed using the HADS subscale score cutoffs defining valid cases [24,27].

The eMindLog Daily scores were subjected to analyses to determine the number and nature of the subscales that exist. The structure of the eMindLog daily items was explored with a maximum likelihood factor analysis with an oblique rotation (SAS Proc Factor, Promax).

## Results

### User Statistics

A total of 198 subjects provided informed consent. One subject provided incomplete data and was excluded. Two subjects’ data were considered invalid—they filled out the scale with a degree of inconsistency that was random or due to illiteracy, and their data were excluded. Thus, data from 195 subjects were analyzed. Software challenges prevented 3 subjects from completing the HADS.

Demographic information is provided in Table 1. The average age was 39.8 (range 18-83), over half (112/195, 57.4%) were female, and over half (115/195, 59.0%) were married or in a committed relationship. Race and ethnicity, education, and economic status are also reported below. The proportion reporting professional care for anxiety/depression was 24.1% (47/195) with 9.7% (19/195) in psychotherapy and 17.9% (35/195) on medications.

**Table 1:** Demographic features of subjects (N=195).

Characteristics		n (%)
Age, mean (SD), range		39.8 (18.66), 18-83
Female		112 (57.4)
**Race**		
	White	139 (71.2)
	African American	41 (21.0)
	Asian	5 (2.6)
	Other	10 (5.1)
**Ethnicity**		
	Hispanic	7 (3.6)
**Marital status**		
	Single	80 (41.0)
	Married/Committed	115 (59.0)
**Education**		
	Attended high school	8 (4.1)
	High school graduate	77 (39.5)
	College graduate	110 (56.4)
	Post-graduate degree	0 (0)
**Economic status**		
	Lower	19 (9.7)
	Middle	149 (76.4)
	Upper	27 (13.8)
Disabled		17 (8.7)
**Receiving professional care for anxiety/depression**		47 (24.1)
	Psychotherapy	19 (9.7)
	Medications	35 (17.9)

**Table 2:** Pearson correlation coefficient between eMindLog daily average total scores with selected eMindLog weekly items (N=195).

Item	Mean (SD)	*r* (*P*)
Stressed	3.84 (2.338)	.60 (<.001)
Restricted quality of life	2.59 (2.625)	.62 (<.001)
Restricted functioning	3.38 (2.764)	.59 (<.001)

The descriptions offered for each item in eMindLog were reported as helpful by 190/194 (97.9%) of the subjects. The daily eMindLog differentiated items into emotions, thoughts, and behaviors. The proportion of subjects who reported being able to differentiate emotions from thoughts was 159/194 (81.5%), and behaviors from emotions/thoughts was 175/194 (90.2%).

The mean eMindLog daily average total score was 1.76 and the median was 1.45. The suicidal thoughts item score had the largest skew (and the only one above 2), with 174 (89%) reporting none (score 0). The daily score was not significantly different between the sexes and racial/ethnic groups. The daily score was negatively correlated with education (*P*<.001) and economic status (*P*=.02). Being single was associated with higher scores than couples (*P*=.002). Those receiving therapy (*P*=.006) and medications (*P*=.03) also had higher scores. Those receiving therapy had a poorer quality of life (*P*=.003) and poorer functioning (*P*=.008), but not those receiving medications.

Only a small number (17/195, 8.7%) of subjects reported being disabled. Disabled individuals had nonsignificantly higher daily scores (*P*=.074). However, disabled individuals reported a poorer quality of life (*P*=.046) and restricted functioning (*P*=.043).

Table 2 reports the correlation between the eMindLog daily total scores and the weekly item scores for “Stressed,” “Restricted Quality of Life,” and “Restricted Function.”. They are each correlated in the 0.6 range (*P*<.001).

Cronbach alpha for eMindLog daily average total and index scores are reported in Table 3. Cronbach alpha was .94 for eMindLog daily score and .86 for the HADS total score. The Social index had a Cronbach alpha of .63, below the .7 acceptable threshold, and thus the questions comprising that index were discarded from further analyses. This reduced the eMindLog daily to 17 questions with 4 indexes (anxiety, anger, sadness, and anhedonia). The other eMindLog index scores had Cronbach alpha in the .81-.83 range.

Pearson correlation coefficients between eMindLog and HADS scores are presented in Table 4. eMindLog anxiety index scores and HADS anxiety subscale score Pearson *r* was .66 (*P*<.001). eMindLog combined sadness and anhedonia index score was correlated with the HADS depression subscale score at *r=*.62 (*P*<.001).

### Diagnostic Thresholds

Table 5 reports the number of subjects (35/195, 17.9%) who met cross-sectional criteria for MDD (29/195, 14.9%) or GAD (23/195, 11.8%). MDD was strongly associated with concurrent GAD (φ=.61, χ^2^_1_=71.80, *P*<.001). Among those with MDD, the odds of also having GAD (1.417) were 37.8 times higher than the odds among those without MDD (.0375). Subjects with GAD or MDD were more likely to be receiving psychotherapy compared to those without (odds ratio 5.19). However, they were not more likely to be receiving medications for anxiety/depression.

**Table 3:** Cronbach alpha for 17-item eMindLog daily and HADS.

	Mean	SD	Range	Cronbach alpha
eMindLog daily total	1.76	1.451	0-7.35	.94
Anxiety index	2.50	1.771	0-7.50	.83
Anger index	1.76	1.831	0-9.00	.82
Sadness index	1.46	1.767	0-8.50	.82
Anhedonia index	1.48	1.494	0-7.33	.81
HADS total	11.5	6.20	0-31	.86
HADS anxiety	7.79	3.79	0-19	.81
HADS depression	3.7	3.25	0-16	.81

**Table 4:** Pearson correlation coefficient for eMindLog daily and HADS scores.

	HADS anxiety, *r* (*P*)	HADS depression, *r* (*P*)
HADS depression	.55 (<.001)	
eMindLog anxiety index	.66 (<.001)	.49 (<.001)
eMindLog sadness & anhedonia indexes	.58 (<.001)	.62 (<.001)

**Table 5:** Subjects meeting eMindLog thresholds for GAD and MDD (N=195).

	GAD, n (%)	No GAD, n (%)	Total, n (%)
MDD	17 (8.7)	12 (6.2)	29 (14.9)
No MDD	6 (3.1)	160 (82.1)	166 (85.1)
Total	23 (11.8)	172 (88.2)	195 (100.0)

A sensitivity analysis performed using binary logistic regression from the HADS subscale scores to evaluate the validity of the algorithms for GAD and MDD were supportive (see Multimedia Appendix 2).

### Factor Analysis

The eMindLog daily scores were subjected to analyses to determine the number and nature of the subscales that exist. The structure of the eMindLog daily items was explored with a maximum likelihood factor analysis with an oblique rotation (SAS Proc Factor, Promax). Much (60%) of the variance in the 17 items was captured in four factors. Tucker and Lewis’ reliability coefficient indicated good fit, with a value of .93. Cluster analysis (SAS, Varclus) was also used to group the 17 items into four clusters. The partitioning of items into clusters matched exactly the partitioning of variables into factors. Cronbach alpha was computed for each of the four subscales. As shown in Table 6, internal consistency/reliability was good, with values ranging from .80-.86.

**Table 6:** Oblique factor analysis of the daily items.

Factor	Item	Greatest beta	Cronbach alpha
1. Anhedonia	14. During the past 24 hours, how lacking in pleasure have you felt?	.73	.86
	15. During the past 24 hours, how lacking in thoughts have you been?	.64	
	17. During the past 24 hours, how lacking in approach have you been?	.62	
	16. During the past 24 hours, how futile have your thoughts been?	.51	
	9. During the past 24 hours, how withdrawn have you been?	.44	
	13. During the past 24 hours, how impulsive have you been?	.39	
2. Sadness	7. During the past 24 hours, how sad have your thoughts been?	.83	.86
	5. During the past 24 hours, how sad have you felt?	.70	
	8. During the past 24 hours, how suicidal have your thoughts been?	.59	
	6. During the past 24 hours, how emotionally numb have you felt?	.52	
3. Anxiety	2. During the past 24 hours, how worried have your thoughts been?	.55	.85
	1. During the past 24 hours, how anxious have you felt?	.48	
	4. During the past 24 hours, how avoidant have you been?	.43	
	3. During the past 24 hours, how physically agitated have you been?	.41	
4. Anger	10. During the past 24 hours, how angry have you felt?	.83	.80
	12. During the past 24 hours, how hostile have you been?	.63	
	11. During the past 24 hours, how blaming have your thoughts been?	.56	

We hypothesized that distinct factors would match the theoretically derived indexes. There were 2 items in the factor/cluster analyses that were discrepant with the theoretically derived index categorizations—the “withdrawal” and “impulsive” items were included in the anhedonia factor. In the theoretically derived index categorization, “withdrawal” falls into the sadness index and “impulsivity” in the anger index.

## Discussion

### Principal Results

eMindLog is a self-measurement tool for tracking anxiety and depression with several unique features. The basis of eMindLog is a hybrid approach that incorporates the clinical perspective and knowledge from the neurosciences *.* It assesses emotions, thoughts, and behaviors relevant to anxiety and depression, as well as associated symptoms, quality of life, and functioning. Subjects reported the ability to differentiate emotions from thoughts (82%) and behaviors from emotions/thoughts (90%). eMindLog minimizes assessment burden by differentiating daily from weekly assessments. eMindLog daily has 17 items reflecting 4 indexes—anxiety, anger, sadness, and anhedonia (lack of pleasure)—as noted previously, the 3 items in the social index were removed as their Cronbach alpha score fell below the .7 threshold. The 3 items were revised based on a re-reading of the relevant literature and moved to the weekly set due to the importance of capturing a measure of interpersonal relationships in assessing anxiety and depression. eMindLog weekly thus has 17 items in 5 domains: associated symptoms, stress, interpersonal, restricted quality of life, and functional impairment. The interpersonal items were not tested in this study but will be particularly scrutinized in future validation studies.

eMindLog enhances measurement precision by describing clearly what the term encompasses and boundaries of an item being assessed, so they are nonoverlapping. Subjects (98%) found value in the items descriptions. eMindLog uses a standard 0 to 10-point scoring system, with descriptive guidance for differentiating severity to enhance accuracy. eMindLog gathers information electronically, using mobile technology to enhance ease of use with dashboard reporting of scores to graphically track scores over time. The individual owns their own data with privacy, confidentiality, and security assured. The user has the ability, if they choose, to share information with their clinician.

This cross-sectional validation study is in a nonclinical, general population convenience sample. The demographics of the study population, including the proportion with clinically significant symptoms, disability, and receiving professional care, support inferential statements from the data. Sex, race, and ethnicity do not impact the eMindLog daily total scores. Higher scores are associated with lower economic status, less education, being single, and receiving professional care. This is consistent with the known adverse effects of low socioeconomic and educational status, and social isolation on mental health. Individuals who reported being disabled (9%) had poorer quality of life and more functional impairment.

eMindLog daily scores had a Cronbach alpha of .94. An alpha score above .7 is considered acceptable for group comparisons for research purposes and an alpha above .9 as supporting reliability for monitoring individual scores [28]. This supports the value of eMindLog for individual patient care in clinical practice.

The daily version of the scale used in this study had 20 questions from which 5 indexes were derived. However, the social index, derived from 3 questions fell below the acceptable Cronbach alpha threshold and the 3 questions and social index were excluded. eMindLog thus has 4 index scores: for anxiety, anger, sadness, and anhedonia, with Cronbach alpha in the .81-.83 range. While the daily total score would suggest the unidimensional nature of the scale, studies in clinical samples are necessary to explore the value of eMindLog index scores in distinguishing biotypes of depression [12,13].

eMindLog index for anxiety had a Pearson correlation coefficient of .66 with the HADS anxiety subscale, and eMindLog sadness/anhedonia indexes with HADS depression subscale was .62. A score above .5 is considered a large convergent validation.

An algorithm derived from the DSM-5 criteria for MDD and GAD suggested that 12% met criteria for GAD and 15% for MDD cross-sectionally. The validity of these algorithms will need to be tested prospectively in studies that use clinician-based diagnoses. These proportions are, however, consistent with what would be expected in the general population. Additionally, the high comorbidity of GAD and MDD is to be expected. GAD and MDD were associated with receiving psychotherapy, but not medication. The more immediate and robust benefits of medications may have obscured demonstrating a relationship here.

Cluster and factor analyses generally support the index structure of the daily eMindLog for anxiety, anger, sadness, and anhedonia. Two items failed to statistically match the theoretically derived formulations. However, this study is in a nonclinical population with the majority of subjects having few symptoms. Hence, such analyses are better performed in data obtained from clinical populations.

eMindLog has unique features. The items measured daily separate emotions, reflective of positive and negative valence systems, from thoughts, derived from cognitive systems. Both drive behavior, the former from bottom-up, and the latter potentially from top-down processing. The neuroscience distinctions of the threat and reward systems guided the indexes, such that the anxiety, anger, and sadness indexes are reflective of functional states of the threat system, while the anhedonia index reflects underactivity of the reward system. Studies of the cognitive system generally focus on standardized batteries assessing attention, working memory, and executive function, but these fail to address the content of thoughts, which are clinically relevant in anxiety and depression. Emotion, thought, and behavior items are weighted proportionately and contribute equally in the construction of index scores, permitting the comparison of index scores with each other. Future studies in clinical populations will examine the validity of these constructs. Each item is described in brief, distinct, nonoverlapping terms, to enhance measurement precision. The symptoms being assessed are placed in a neuroscience framework—thus, unlike some scales that measure items such as hopeless and worthless thoughts independently, enhancing the influence of thoughts in the total score, eMindLog asks the user to capture all such thoughts in a single item called sad thoughts. Guidance is provided to the user in choosing a score reflective of their experience [25]. Assessment burden is taken into consideration, so that ancillary symptoms needed for imputing diagnostic thresholds are not assessed daily, but every 7 days. Quality of life and functioning are measured separately, since these may provide a different perspective from symptom measures.

### Limitations

While eMindLog has features that positively differentiate it from other self-report measures in anxiety and depression, there are still limitations to be acknowledged. To reflect the flow of mental life, eMindLog entails the daily self-assessment of emotions, thoughts, and behaviors. The average time in the study to score the daily eMindLog was 7.3 minutes and 6.0 minutes for the weekly eMindLog; subjects were using this measure for the first time. Repeat eMindLog users report a time commitment around 3 minutes for each of the daily and weekly components. However, this still requires effort and ongoing adherence. The degree of adherence necessary to provide a minimum threshold for appropriate signal detection remains to be explored.

Somatic components of anxiety and depression are minimally assessed in eMindLog. “Lack of well-being,” defined as generally feeling ill or unwell, is the only item that addresses somatic symptoms. Somatic symptoms can either reflect emotional expression or a general medical condition. Such a distinction is difficult to make, and whether it can be made accurately and reliably in a self-report measure without a clinician’s judgment, is uncertain.

eMindLog requires a level of sophisticated awareness of subjective processes, breaking down complex experiences into component parts. The precision of the measure is reported by users to be enhanced with practice and experience. An initial practice period is useful for the maturing of self-observation and while terms and their descriptions are harmonized with a user’s subjective experiences. Data from further validation studies, particularly in clinical populations, are needed to explore whether eMindLog has superior signal detection properties than alternatives.

eMindLog has the promise of using mobile technology to gather data that reflect the richness of mental phenomena for use in case detection, monitoring severity, evaluating therapeutic interventions, and clinical trials. Confirmation in clinical populations, particularly the ability to separate the ill from the non-ill is essential. Additional studies will need to explore the responsiveness to interventions with adequate precision and to document delineations for remission in anxiety and depressive disorders.

While eMindLog demonstrates strong unidimensional characteristics in this validation study, only studies in clinical populations with different diagnoses and in those with general medical conditions, can examine whether index scores have differential utility in specific populations. If eMindLog demonstrates enhanced measurement precision, it may be a useful tool for drug development in anxiety and depression. Enhanced measurement precision may also assist in the discovery of disease biomarkers.

### Conclusions

A novel self-measure of anxiety and depression using mobile technology, is presented. Data from a validation study of eMindLog in the general population demonstrates excellent reliability and a large convergent validity against a standard measure. Future studies in clinical populations will provide an assessment of the full potential for eMindLog.

## Appendix

Multimedia Appendix 1 Screenshots of website used in the study.

Multimedia Appendix 2 Sensitivity analysis of diagnostic algorithms using Hospital Anxiety and Depression Scale (HADS).

## Abbreviations

DSM-5: Diagnostic and Statistical Manual of Mental Disorders, Fifth Edition
GAD: Generalized Anxiety Disorder
HADS: Hospital Anxiety and Depression Scale
MDD: Major Depressive Disorder
RDoC: Research Domain Criteria
