# Supplementary material for: eMindLog: Self-Measurement of Anxiety and Depression Using Mobile Technology
Source: JMIR Res Protoc. 2017 May 24;6(5):e98. doi: 10.2196/resprot.7447 (PMC5463054; doi:10.2196/resprot.7447)
Supplement: Multimedia Appendix 1 [file resprot_v6i5e98_app1.pdf]

MULTIMEDIA APPENDIX

This is a Multimedia Appendix to a full manuscript published in the J Med Internet Res, for full copyright and citation information see http://www.researchprotocols.org/0000/0/e0/
doi:10.2196/resprot.7447

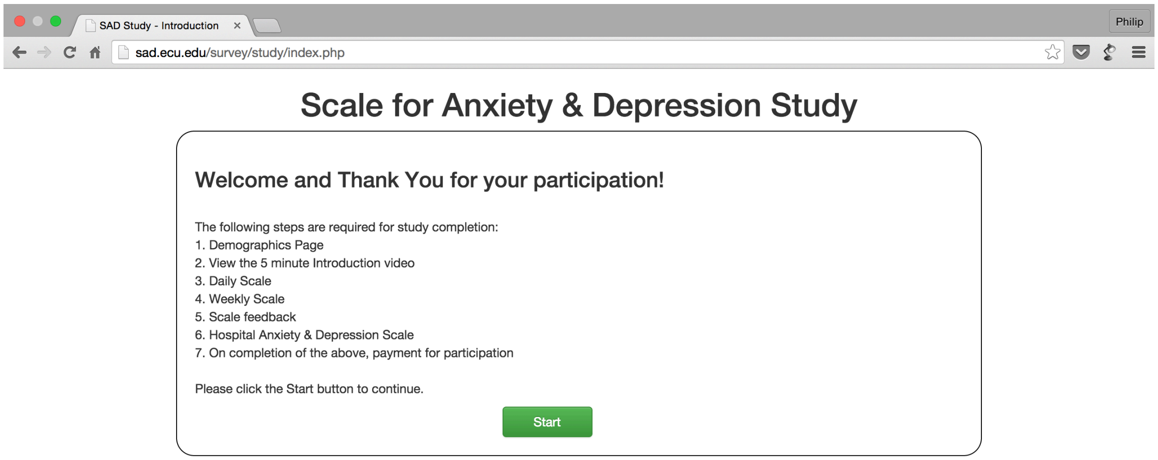

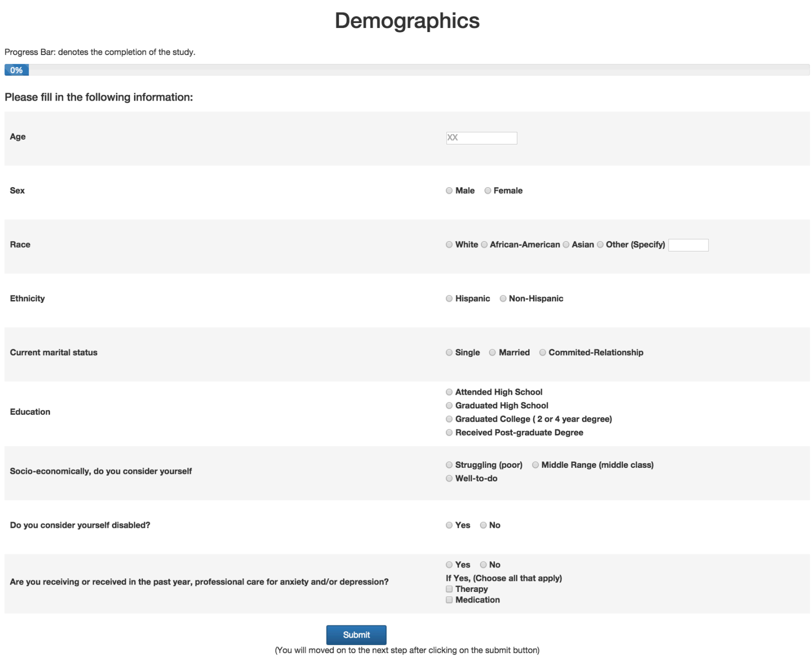

Demographics

Progress Bar: denotes the completion of the study.

0%

Please fill in the following information:

**Age**	XX
**Sex**	Male Female
**Race**	White African-American Asian Other (Specify)
**Ethnicity**	Hispanic Non-Hispanic
**Current marital status**	Single Married Commited-Relationship
**Education**	Attended High School<br>Graduated High School<br>Graduated College (2 or 4 year degree)<br>Received Post-graduate Degree
**Socio-economically, do you consider yourself**	Struggling (poor) Middle Range (middle class)<br>Well-to-do
**Do you consider yourself disabled?**	Yes No
**Are you receiving or received in the past year, professional care for anxiety and/or depression?**	Yes No<br>If Yes, (Choose all that apply)<br>Therapy<br>Medication

Submit

(You will moved on to the next step after clicking on the submit button)

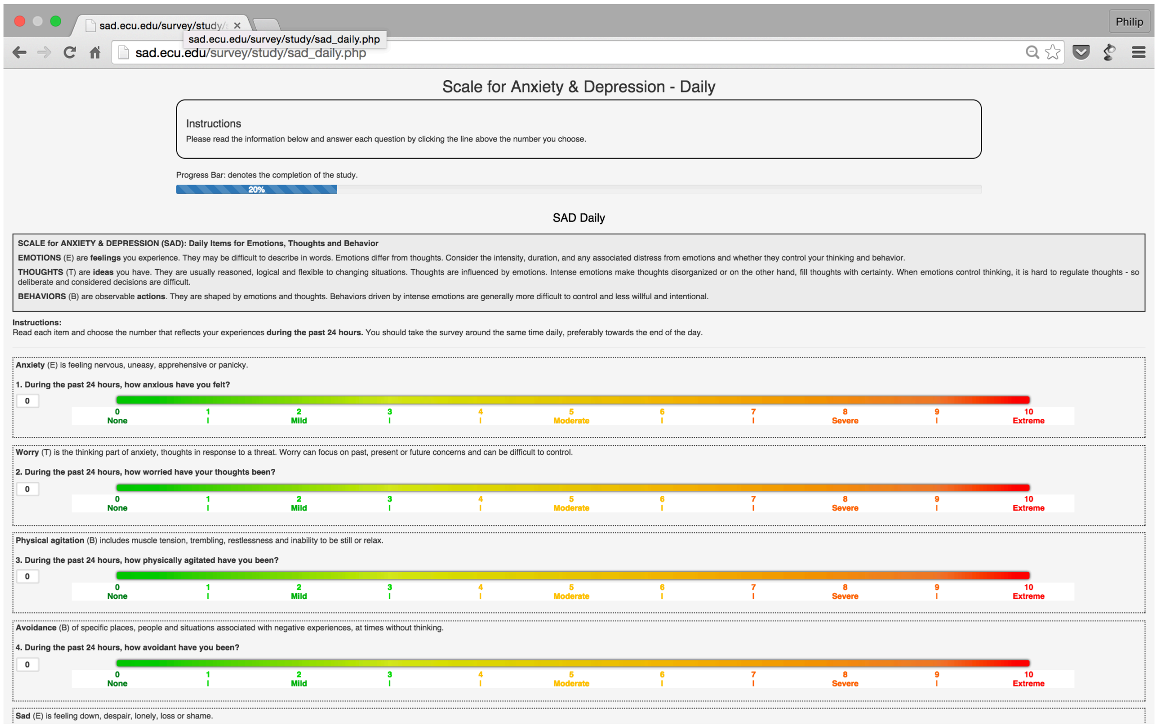

sad.ecu.edu/survey/study/ ×

Philip

sad.ecu.edu/survey/study/sad_daily.php

sad.ecu.edu/survey/study/sad_daily.php

Scale for Anxiety & Depression - Daily

Instructions

Please read the information below and answer each question by clicking the line above the number you choose.

Progress Bar: denotes the completion of the study.

20%

SAD Daily

SCALE for ANXIETY & DEPRESSION (SAD): Daily Items for Emotions, Thoughts and Behavior

EMOTIONS (E) are **feelings** you experience. They may be difficult to describe in words. Emotions differ from thoughts. Consider the intensity, duration, and any associated distress from emotions and whether they control your thinking and behavior.

THOUGHTS (T) are **ideas** you have. They are usually reasoned, logical and flexible to changing situations. Thoughts are influenced by emotions. Intense emotions make thoughts disorganized or on the other hand, fill thoughts with certainty. When emotions control thinking, it is hard to regulate thoughts - so deliberate and considered decisions are difficult.

BEHAVIORS (B) are observable **actions**. They are shaped by emotions and thoughts. Behaviors driven by intense emotions are generally more difficult to control and less willful and intentional.

Instructions:
Read each item and choose the number that reflects your experiences **during the past 24 hours.** You should take the survey around the same time daily, preferably towards the end of the day.

Anxiety (E) is feeling nervous, uneasy, apprehensive or panicky.

1. During the past 24 hours, how anxious have you felt?

0

0	1	2	3	4	5	6	7	8	9	10
None	I	Mild	I	I	Moderate	I	I	Severe	I	Extreme

Worry (T) is the thinking part of anxiety, thoughts in response to a threat. Worry can focus on past, present or future concerns and can be difficult to control.

2. During the past 24 hours, how worried have your thoughts been?

0

0	1	2	3	4	5	6	7	8	9	10
None	I	Mild	I	I	Moderate	I	I	Severe	I	Extreme

Physical agitation (B) includes muscle tension, trembling, restlessness and inability to be still or relax.

3. During the past 24 hours, how physically agitated have you been?

0

0	1	2	3	4	5	6	7	8	9	10
None	I	Mild	I	I	Moderate	I	I	Severe	I	Extreme

Avoidance (B) of specific places, people and situations associated with negative experiences, at times without thinking.

4. During the past 24 hours, how avoidant have you been?

0

0	1	2	3	4	5	6	7	8	9	10
None	I	Mild	I	I	Moderate	I	I	Severe	I	Extreme

Sad (E) is feeling down, despair, lonely, loss or shame.

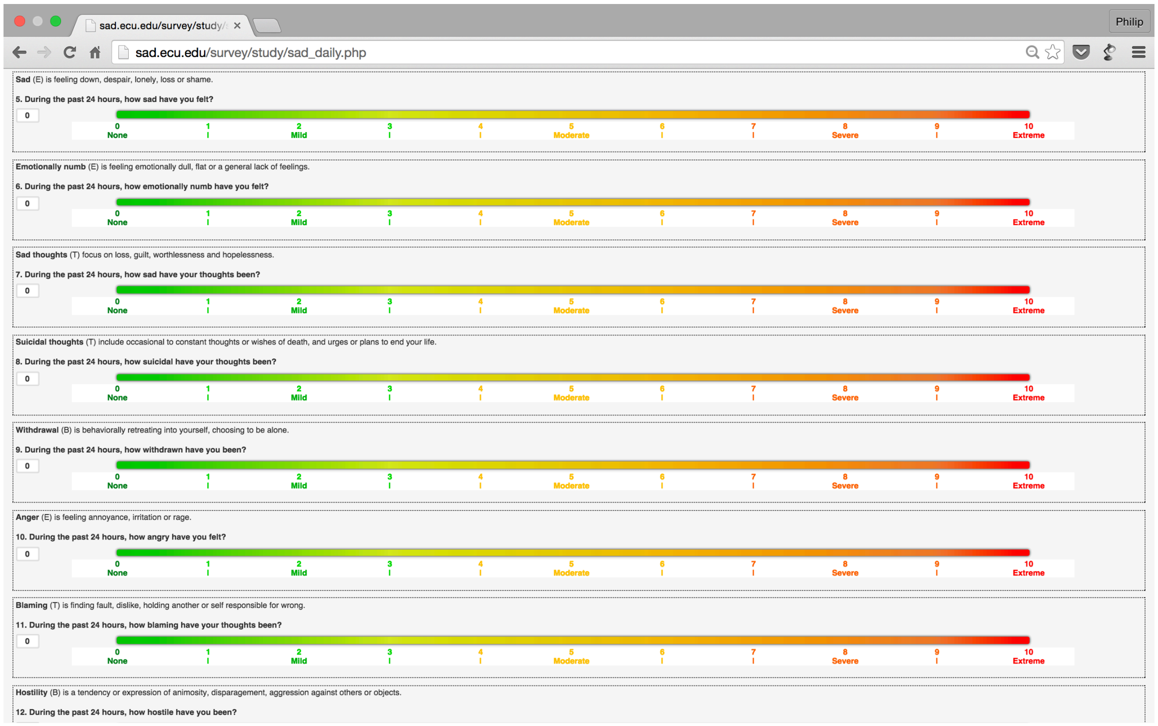

sad.ecu.edu/survey/study/ ×

Philip

sad.ecu.edu/survey/study/sad_daily.php

Sad (E) is feeling down, despair, lonely, loss or shame.

5. During the past 24 hours, how sad have you felt?

0

0 None | 1 | 2 Mild | 3 | 4 | 5 Moderate | 6 | 7 | 8 Severe | 9 | 10 Extreme

Emotionally numb (E) is feeling emotionally dull, flat or a general lack of feelings.

6. During the past 24 hours, how emotionally numb have you felt?

0

0 None | 1 | 2 Mild | 3 | 4 | 5 Moderate | 6 | 7 | 8 Severe | 9 | 10 Extreme

Sad thoughts (T) focus on loss, guilt, worthlessness and hopelessness.

7. During the past 24 hours, how sad have your thoughts been?

0

0 None | 1 | 2 Mild | 3 | 4 | 5 Moderate | 6 | 7 | 8 Severe | 9 | 10 Extreme

Suicidal thoughts (T) include occasional to constant thoughts or wishes of death, and urges or plans to end your life.

8. During the past 24 hours, how suicidal have your thoughts been?

0

0 None | 1 | 2 Mild | 3 | 4 | 5 Moderate | 6 | 7 | 8 Severe | 9 | 10 Extreme

Withdrawal (B) is behaviorally retreating into yourself, choosing to be alone.

9. During the past 24 hours, how withdrawn have you been?

0

0 None | 1 | 2 Mild | 3 | 4 | 5 Moderate | 6 | 7 | 8 Severe | 9 | 10 Extreme

Anger (E) is feeling annoyance, irritation or rage.

10. During the past 24 hours, how angry have you felt?

0

0 None | 1 | 2 Mild | 3 | 4 | 5 Moderate | 6 | 7 | 8 Severe | 9 | 10 Extreme

Blaming (T) is finding fault, dislike, holding another or self responsible for wrong.

11. During the past 24 hours, how blaming have your thoughts been?

0

0 None | 1 | 2 Mild | 3 | 4 | 5 Moderate | 6 | 7 | 8 Severe | 9 | 10 Extreme

Hostility (B) is a tendency or expression of animosity, disparagement, aggression against others or objects.

12. During the past 24 hours, how hostile have you been?

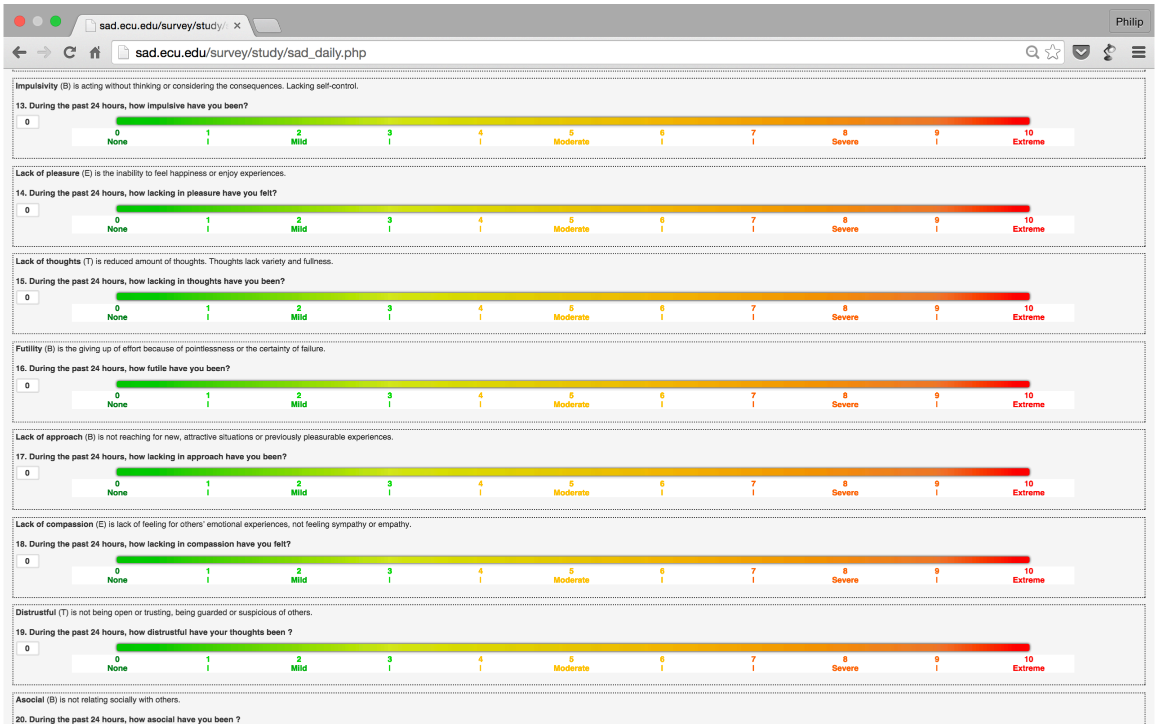

sad.ecu.edu/survey/study/ ×

Philip

sad.ecu.edu/survey/study/sad_daily.php

Impulsivity (B) is acting without thinking or considering the consequences. Lacking self-control.

13. During the past 24 hours, how impulsive have you been?

0

0 None | 1 | 2 Mild | 3 | 4 | 5 Moderate | 6 | 7 | 8 Severe | 9 | 10 Extreme

Lack of pleasure (E) is the inability to feel happiness or enjoy experiences.

14. During the past 24 hours, how lacking in pleasure have you felt?

0

0 None | 1 | 2 Mild | 3 | 4 | 5 Moderate | 6 | 7 | 8 Severe | 9 | 10 Extreme

Lack of thoughts (T) is reduced amount of thoughts. Thoughts lack variety and fullness.

15. During the past 24 hours, how lacking in thoughts have you been?

0

0 None | 1 | 2 Mild | 3 | 4 | 5 Moderate | 6 | 7 | 8 Severe | 9 | 10 Extreme

Futility (B) is the giving up of effort because of pointlessness or the certainty of failure.

16. During the past 24 hours, how futile have you been?

0

0 None | 1 | 2 Mild | 3 | 4 | 5 Moderate | 6 | 7 | 8 Severe | 9 | 10 Extreme

Lack of approach (B) is not reaching for new, attractive situations or previously pleasurable experiences.

17. During the past 24 hours, how lacking in approach have you been?

0

0 None | 1 | 2 Mild | 3 | 4 | 5 Moderate | 6 | 7 | 8 Severe | 9 | 10 Extreme

Lack of compassion (E) is lack of feeling for others' emotional experiences, not feeling sympathy or empathy.

18. During the past 24 hours, how lacking in compassion have you felt?

0

0 None | 1 | 2 Mild | 3 | 4 | 5 Moderate | 6 | 7 | 8 Severe | 9 | 10 Extreme

Distrustful (T) is not being open or trusting, being guarded or suspicious of others.

19. During the past 24 hours, how distrustful have your thoughts been ?

0

0 None | 1 | 2 Mild | 3 | 4 | 5 Moderate | 6 | 7 | 8 Severe | 9 | 10 Extreme

Asocial (B) is not relating socially with others.

20. During the past 24 hours, how asocial have you been ?

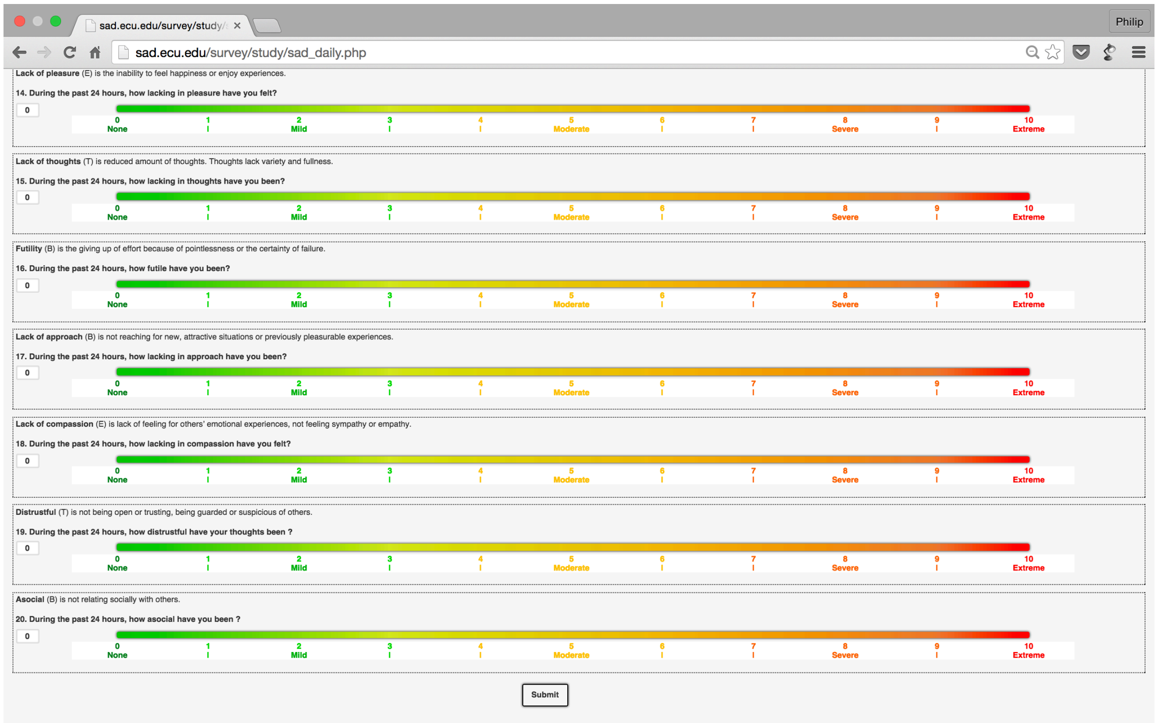

sad.ecu.edu/survey/study/ ×

Philip

sad.ecu.edu/survey/study/sad_daily.php

Lack of pleasure (E) is the inability to feel happiness or enjoy experiences.

14. During the past 24 hours, how lacking in pleasure have you felt?

0

0 None | 1 | 2 Mild | 3 | 4 | 5 Moderate | 6 | 7 | 8 Severe | 9 | 10 Extreme

Lack of thoughts (T) is reduced amount of thoughts. Thoughts lack variety and fullness.

15. During the past 24 hours, how lacking in thoughts have you been?

0

0 None | 1 | 2 Mild | 3 | 4 | 5 Moderate | 6 | 7 | 8 Severe | 9 | 10 Extreme

Futility (B) is the giving up of effort because of pointlessness or the certainty of failure.

16. During the past 24 hours, how futile have you been?

0

0 None | 1 | 2 Mild | 3 | 4 | 5 Moderate | 6 | 7 | 8 Severe | 9 | 10 Extreme

Lack of approach (B) is not reaching for new, attractive situations or previously pleasurable experiences.

17. During the past 24 hours, how lacking in approach have you been?

0

0 None | 1 | 2 Mild | 3 | 4 | 5 Moderate | 6 | 7 | 8 Severe | 9 | 10 Extreme

Lack of compassion (E) is lack of feeling for others' emotional experiences, not feeling sympathy or empathy.

18. During the past 24 hours, how lacking in compassion have you felt?

0

0 None | 1 | 2 Mild | 3 | 4 | 5 Moderate | 6 | 7 | 8 Severe | 9 | 10 Extreme

Distrustful (T) is not being open or trusting, being guarded or suspicious of others.

19. During the past 24 hours, how distrustful have your thoughts been ?

0

0 None | 1 | 2 Mild | 3 | 4 | 5 Moderate | 6 | 7 | 8 Severe | 9 | 10 Extreme

Asocial (B) is not relating socially with others.

20. During the past 24 hours, how asocial have you been ?

0

0 None | 1 | 2 Mild | 3 | 4 | 5 Moderate | 6 | 7 | 8 Severe | 9 | 10 Extreme

Submit

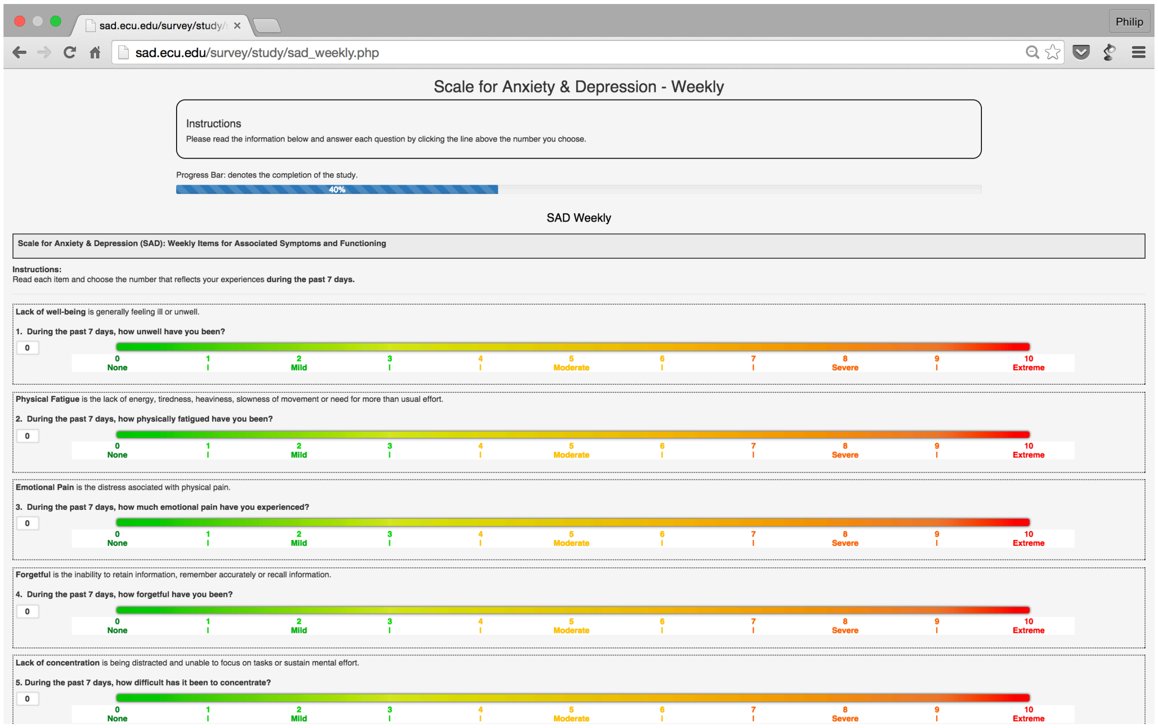

sad.ecu.edu/survey/study/ ×

Philip

sad.ecu.edu/survey/study/sad_weekly.php

Scale for Anxiety & Depression - Weekly

Instructions

Please read the information below and answer each question by clicking the line above the number you choose.

Progress Bar: denotes the completion of the study.

40%

SAD Weekly

Scale for Anxiety & Depression (SAD): Weekly Items for Associated Symptoms and Functioning

Instructions:
Read each item and choose the number that reflects your experiences **during the past 7 days.**

Lack of well-being is generally feeling ill or unwell.

1. During the past 7 days, how unwell have you been?

0

0 None | 1 | 2 Mild | 3 | 4 | 5 Moderate | 6 | 7 | 8 Severe | 9 | 10 Extreme

Physical Fatigue is the lack of energy, tiredness, heaviness, slowness of movement or need for more than usual effort.

2. During the past 7 days, how physically fatigued have you been?

0

0 None | 1 | 2 Mild | 3 | 4 | 5 Moderate | 6 | 7 | 8 Severe | 9 | 10 Extreme

Emotional Pain is the distress associated with physical pain.

3. During the past 7 days, how much emotional pain have you experienced?

0

0 None | 1 | 2 Mild | 3 | 4 | 5 Moderate | 6 | 7 | 8 Severe | 9 | 10 Extreme

Forgetful is the inability to retain information, remember accurately or recall information.

4. During the past 7 days, how forgetful have you been?

0

0 None | 1 | 2 Mild | 3 | 4 | 5 Moderate | 6 | 7 | 8 Severe | 9 | 10 Extreme

Lack of concentration is being distracted and unable to focus on tasks or sustain mental effort.

5. During the past 7 days, how difficult has it been to concentrate?

0

0 None | 1 | 2 Mild | 3 | 4 | 5 Moderate | 6 | 7 | 8 Severe | 9 | 10 Extreme

sad.ecu.edu/survey/study/ ×

Philip

sad.ecu.edu/survey/study/sad_weekly.php

Disturbed appetite is a disruption in the desire and ability to eat appropriately (i.e. increase, decrease, change in diet or schedule) and being satisfied with food.

6. During the past 7 days, how disturbed has your appetite been?

0

0 None | 1 | 2 Mild | 3 | 4 | 5 Moderate | 6 | 7 | 8 Severe | 9 | 10 Extreme

Disturbed sleep includes difficulty falling asleep in a reasonable time, staying asleep, oversleeping and/or not feeling refreshed on waking.

7. During the past 7 days, how disturbed has your sleep been?

0

0 None | 1 | 2 Mild | 3 | 4 | 5 Moderate | 6 | 7 | 8 Severe | 9 | 10 Extreme

Stressed is being overwhelmed, distraught, vulnerable and not in control.

8. During the past 7 days, how stressed have you been?

0

0 None | 1 | 2 Mild | 3 | 4 | 5 Moderate | 6 | 7 | 8 Severe | 9 | 10 Extreme

Quality of life is fulfillment and contentment in your life.

9. During the past 7 days, how restricted has the quality of your life been?

0

0 None | 1 | 2 Mild | 3 | 4 | 5 Moderate | 6 | 7 | 8 Severe | 9 | 10 Extreme

Social Function is the ability to fulfill expectations in your social role.

10.1 During the past 7 days, how restricted in your *social* functioning have you been?

0

0 None | 1 | 2 Mild | 3 | 4 | 5 Moderate | 6 | 7 | 8 Severe | 9 | 10 Extreme

Work Function is the ability to fulfill expectations in your role at work. If not working, choose 0.

10.2 During the past 7 days, how restricted in your *work* functioning have you been?

0

0 None | 1 | 2 Mild | 3 | 4 | 5 Moderate | 6 | 7 | 8 Severe | 9 | 10 Extreme

School Function is the ability to fulfill expectations in your role at school. If not in school, choose 0.

10.3 During the past 7 days, how restricted in your *school* functioning have you been?

0

0 None | 1 | 2 Mild | 3 | 4 | 5 Moderate | 6 | 7 | 8 Severe | 9 | 10 Extreme

Home Function is the ability to fulfill expectations in your role at home.

10.4 During the past 7 days, how restricted in your *home* functioning have you been?

sad.ecu.edu/survey/study/ ×

Philip

sad.ecu.edu/survey/study/sad_weekly.php

0

0 None | 1 | 2 Mild | 3 | 4 | 5 Moderate | 6 | 7 | 8 Severe | 9 | 10 Extreme

Quality of life is fulfillment and contentment in your life.

9. During the past 7 days, how restricted has the quality of your life been?

0

0 None | 1 | 2 Mild | 3 | 4 | 5 Moderate | 6 | 7 | 8 Severe | 9 | 10 Extreme

Social Function is the ability to fulfill expectations in your social role.

10.1 During the past 7 days, how restricted in your *social* functioning have you been?

0

0 None | 1 | 2 Mild | 3 | 4 | 5 Moderate | 6 | 7 | 8 Severe | 9 | 10 Extreme

Work Function is the ability to fulfill expectations in your role at work. If not working, choose 0.

10.2 During the past 7 days, how restricted in your *work* functioning have you been?

0

0 None | 1 | 2 Mild | 3 | 4 | 5 Moderate | 6 | 7 | 8 Severe | 9 | 10 Extreme

School Function is the ability to fulfill expectations in your role at school. If not in school, choose 0.

10.3 During the past 7 days, how restricted in your *school* functioning have you been?

0

0 None | 1 | 2 Mild | 3 | 4 | 5 Moderate | 6 | 7 | 8 Severe | 9 | 10 Extreme

Home Function is the ability to fulfill expectations in your role at home.

10.4 During the past 7 days, how restricted in your *home* functioning have you been?

0

0 None | 1 | 2 Mild | 3 | 4 | 5 Moderate | 6 | 7 | 8 Severe | 9 | 10 Extreme

Personal Grooming/Hygiene Function is the ability to fulfill expectations in your personal grooming/hygiene function.

10.5 During the past 7 days, how restricted in your *personal grooming/hygiene* functioning have you been?

0

0 None | 1 | 2 Mild | 3 | 4 | 5 Moderate | 6 | 7 | 8 Severe | 9 | 10 Extreme

Submit

Â© 2014 East Carolina University

SAD Study - Feedback

SAD Study - Feedback ey/study/feedback.php

Philip

Feedback on the Scale for Anxiety & Depression

Instructions

In this section, you are asked to provide feedback on the Scale for Anxiety & Depression.

Progress Bar: denotes the completion of the study.

60%

Please fill in the following form. All the fields are required.

**How many minutes do you estimate it took you to fill out**	
**Daily questions in the Scale for Anxiety & Depression**	XX
**Weekly questions in the Scale for Anxiety & Depression**	XX
**Do you believe the Scale for Anxiety & Depression is useful?**	Yes No
**Would you use the Scale for Anxiety and Depression ?**	Yes No
**Would you use the Scale for Anxiety and Depression if it were available on the internet?**	Yes No
**Were the descriptions of the items helpful?**	Yes No
**Can you generally separate emotions from thoughts?**	Yes No
**Can you generally separate behavior from emotions and thoughts?**	Yes No
**What would you be willing to pay monthly for use of the Scale for Anxiety & Depression?**	$0 $1 $2 $3 $5 $10 More than $10

Submit

(You will moved on to the next step after clicking on the submit button)
