# Supplementary material for: eMindLog: Self-Measurement of Anxiety and Depression Using Mobile Technology
Source: JMIR Res Protoc. 2017 May 24;6(5):e98. doi: 10.2196/resprot.7447 (PMC5463054; doi:10.2196/resprot.7447)
Supplement: Multimedia Appendix 2 [file resprot_v6i5e98_app2.pdf]

Multimedia Appendix 2. Sensitivity Analysis of Diagnostic Algorithms using Hospital Anxiety and Depression Scale (HADS)

Binary logistic regression was used to relate the eMindLog-derived GAD and MDD, to scores from the HADS-Anxiety and HADS-Depression subscale scores. Zigmond and Snaith [1] recommended a cutoff of 8 for borderline cases and 11 for valid cases. Bjelland, Dahl, Haug, and Necklemann [2] noted that 8 is the optimal cutoff scores for studies conducted in the general community. Both these cutoffs were employed in a logistic regression.

Anxiety: Using a cutoff of 8, 96 of the 192 (50%) subjects were categorized as having at least 'borderline anxiety'. The regression predicting 'borderline anxiety' from the eMindLog indexes (Anxiety, Anger, and Anhedonia/Sadness) was significant, but only Anxiety had a significant unique effect. So Anger and Anhedonia/Sadness were dropped from the model. The reduced model was significant, $\chi^2(1)=59.39$, $P<.001$, *OR* 2.18, sensitivity=.77, specificity=.65, *AUC*=.80. Using a cutoff of 11 for valid cases, 43 of 192 (22%) subjects were categorized with 'anxiety'. Anhedonia/Sadness did not have a significant effect, but a model containing Anxiety and Anger was significant $\chi^2(2)=80.43$, $P<.001$, sensitivity=.84, specificity=.80, *AUC*=.90, with both Anxiety, $\chi^2(1)=26.91$, $P<.001$, *OR* 2.58, and Anger, $\chi^2(1)=5.14$, $P=.023$, *OR* 1.34, having significant unique effects.

Depression: Using a cutoff of 8, 27 of 192 (14%) subjects qualified for a category of at least 'borderline depression'. Only Anhedonia/Sadness had a significant effect, $\chi^2(1)=34.18$, $P<.001$, *OR* 2.15, sensitivity=.78, specificity=.70, *AUC*=.82. Using a cutoff of 11 for valid cases, 10 of 192 (5%) subjects were categorized as having 'depression'. Only Anhedonia/Sadness had a significant effect, $\chi^2(1)=25.70$, $P<.001$, *OR* 2.56, sensitivity=.90, specificity=.86, *AUC*=.94.

1. Zigmond AS, Snaith RP. The hospital anxiety and depression scale. Acta Psychiatr Scand. 1983 Jun;67(6):361-70. PMID:6880820
2. Bjelland I, Dahl AA, Haug TT, Neckelmann D. The validity of the Hospital Anxiety and Depression Scale. An updated literature review. J Psychosom Res. 2002 Feb;52(2):69-77. Review. PMID:11832252
